# Real-Time Distributed Architecture for Remote Acoustic Elderly Monitoring in Residential-Scale Ambient Assisted Living Scenarios

**DOI:** 10.3390/s18082492

**Published:** 2018-08-01

**Authors:** Joan Navarro, Ester Vidaña-Vila, Rosa Ma Alsina-Pagès, Marcos Hervás

**Affiliations:** 1 GRITS—Grup de Recerca en Internet Techologies and Storage, c/Quatre Camins, 30, 08022 Barcelona, Spain; 2 GTM—Grup de Recerca en Tecnologies Mèdia, c/Quatre Camins, 30, 08022 Barcelona, Spain; ester.vidana@salle.url.edu (E.V.-V.); rosamaria.alsina@salle.url.edu (R.M.A.-P.); 3 FICOSA—Can Mitjans, s/n, 08232 Viladecavalls, Barcelona, Spain; marcosantonio.hervas@salle.url.edu

**Keywords:** ambient assisted living, acoustic sensor network, graphics processor unit, home monitoring, residence assistance, surveillance

## Abstract

Ambient Assisted Living (AAL) has become a powerful alternative to improving the life quality of elderly and partially dependent people in their own living environments. In this regard, tele-care and remote surveillance AAL applications have emerged as a hot research topic in this domain. These services aim to infer the patients’ status by means of centralized architectures that collect data from a set of sensors deployed in their living environment. However, when the size of the scenario and number of patients to be monitored increase (e.g., residential areas, retirement homes), these systems typically struggle at processing all associated data and providing a reasonable output in real time. The purpose of this paper is to present a fog-inspired distributed architecture to collect, analyze and identify up to nine acoustic events that represent abnormal behavior or dangerous health conditions in large-scale scenarios. Specifically, the proposed platform collects data from a set of wireless acoustic sensors and runs an automatic two-stage audio event classification process to decide whether or not to trigger an alarm. Conducted experiments over a labeled dataset of 7116 s based on the priorities of the Fundació Ave Maria health experts have obtained an overall accuracy of 94.6%.

## 1. Introduction

Human life expectancy is increasing in modern society, and it will continue to do so throughout the next century [1]. There is a strong economic reason for governments to empower both elderly and partially dependent people to live by themselves or at least, with the minimum caring services required. This would minimize costs and improve elderly people’s independent life. In this regard, Ambient Assisted Living (AAL) systems [2] have become a very popular strategy to adapt the patients’ living environments to their specific needs, which improves life quality and optimizes healthcare resources [3]. AAL applications range from health monitoring (e.g., heart rate, body temperature, stroke) to patient surveillance (e.g., dementia, falls), including environment monitoring (e.g., fire, flooding). Typically, these AAL systems combine a Wireless Sensor Network (WSN) composed of hardware with low power consumption, aimed at sensing the patient status, with a data analysis system, aimed at inferring the patient status from the collected data.

Unlike any other sensory system, Wireless Acoustic Sensor Networks (WASN) have proven to be a good solution to enable older patients to interact with AAL solutions [4] because they (1) are easy to deploy in any environment and (2) can serve multiple purposes, from measuring the acoustic activity for surveillance services [5], to enhancing the robustness of acoustic detection systems [6] or even monitoring the behavior of the patients [7]. Acoustic sensing has a large number of applications also in environmental monitoring and surveillance. Actually, acoustic sensor measurements are being deployed in several recent projects in urban scenarios in order to improve health and quality of life in cities. Some of them use WASNs (see [8] or [9]) to monitor the road traffic noise in urban scenarios, and others prefer to invite citizens to contribute with their measurements [10], in which they even teach students to learn acoustics with the measurements. One of the pioneers in that field was project Noisetube [11], where GPS-equipped mobile phones were used to measure the noise pollution. In this sense, it is important to remark that noise pollution has become a major health problem, with several adverse effects: sleep disorders with awakenings [12], learning impairment [13,14], hypertension ischemic heart disease [15] and especially annoyance [16]. There are several public buildings—schools, hospitals and any assisted living scenarios—where these effects can be even more severe because the inhabitants are more sensitive and vulnerable [17].

Home monitoring is especially targeted at those patients who have a high degree of autonomy [3], but need to be continuously tracked due to their age or illness [18]. The privacy of the patients and the cost are two crucial issues to take into account when designing any AAL-based system to supervise a broad amount of people. In this sense, acoustic smart AAL technologies present suitable solutions to provide a minimally-intrusive emergency detection in both home and residential environments [2]. A sound example of this situation can be found at the Fundació Ave Maria residential area (FAM). FAM is a non-profit organization that offers specialized attention to adults with intellectual disabilities. Of all the services that FAM offers, two of them are key issues in this project. The first one is the management of a residential campus for people with intellectual disabilities needing total support. The second one is a network of homes in the village with direct supervision. They offer attention 24 hours a day, seven days a week. The network of homes in the village is devoted to adults with disabilities who want to live with autonomy, needing low-intensity intermittent services.

So far, successful existing AAL approaches have generally been deployed in controlled and low-scale indoor environments [19,20,21,22,23]. Despite the great advances achieved in this domain in terms of individual autonomy, these approaches still restrict the patient movements in a reduced area (e.g., a room or a flat at most). In fact, to the best of our knowledge, very few attempts have been made to conceive of a large-scale AAL system able to monitor patients all over a diverse residential area such as the one managed by the FAM.

The challenges of building an AAL system for large-scale scenarios are two-fold. On the one hand, the amount of data to be processed grows according to the physical surface and/or number of patients to be monitored, which then requires powerful devices to process and store all the sensed information in a reasonable response time [24]. On the other hand, all these data that sensors, patients and medical supervisors continuously exchange must navigate through heterogeneous communication networks and technologies (e.g., BlueTooth, Wi-Fi, Internet) that typically struggle when attempting to meet the stringent Quality of Service (QoS) levels (i.e., bandwidth, reliability and delay) required by AAL applications [25]. However, the latest advances in distributed systems (i.e., fog computing, parallel computing) and data management (i.e., data mining, cloud computing) may greatly contribute to extending current achievements in AAL to large-scale scenarios.

The purpose of this paper is to present the development of a distributed infrastructure to conduct acoustic event recognition accurately in residential environments in order to support independent aging [26]. More specifically, acoustic samples are acquired and processed by means of a fog computing architecture, automatically classified by means of a neural network running in a General Purpose Graphics Processing Unit (GPGPU) and delivered to a high-level decision support system to trigger user-defined alarms when necessary. Hence, the proposed system combines a Wireless Acoustic Sensor Network (WASN), composed of several microphones deployed in a residential area, with a fog computing architecture to identify up to nine different environmental sounds related to AAL events in real time.

The proposed platform is inspired by the homeSound project [23], which was conceived as a first approach to AAL support in indoor environments. The hardware core of homeSound was also composed of a low-cost GPGPU platform [27] that was able to identify up to 14 different acoustic events (e.g., water, walking, glass breaking, dog barking, etc.). The proposal presented in this paper improves the homeSound platform by (1) extending the monitoring range to residential areas, (2) focusing on patient-centered acoustic events and (3) building an intelligent two-layer data analysis system to improve the reliability and accuracy of the acoustic event detection system.

Specifically, the main contributions of this work are the following. First is a review of the latest advances of existing AAL research projects, how they can benefit from WASNs and how fog computing systems are being used to address existing limitations of AAL deployments larger than domestic indoor environments. Second is the conception of a WASN deployed on top of a scalable distributed architecture, inspired by the fog computing paradigm, able to provide AAL support in residential areas. Third is an intelligent data analysis system that combines an artificial neural network with a Case-Based Reasoning (CBR) system to reliably identify acoustic events in real-time.

The remainder of this paper is organized as follows. Section 2 reviews the state of the art of AAL projects regarding WASN and fog computing architectures. Section 3 details the FAM scenario and presents the environment and requirements where the AAL platform will be deployed. Section 4 details the proposed system architecture including the fog computing layout, the sensors deployment and the acoustic nature of the events to be detected. Section 5 details the automatic acoustic data classification system. Next, in Section 6, a discussion of the main constraints of the problem, as well as the preliminary classification results and relevant aspects of the proposed solution is conducted. Finally, Section 7 outlines the main conclusions of this work.

## 2. Related Work

Recent advances in research and technology have broadened the horizons of the AAL platforms committed to assisting aging adults in their own residences. Definitely, WASNs have played a crucial role in the development of non-invasive strategies to monitor people in indoor environments. Additionally, fog computing architectures have contributed to reducing the cost and requirements of surveillance platforms by offloading the heavy computation and storage tasks associated with healthcare monitoring to the cloud. The purpose of this section is two-fold. On the one hand, the contributions to the areas of AAL research projects, WASNs for tele-care applications and fog computing for health monitoring are reviewed. On the other hand, the limitations of the homeSound platform [23] when addressing AAL in a residential area are discussed.

### 2.1. Ambient Assisted Living Research Projects

The concept of the smart home is fundamental for any AAL project design. It is usually described as a regular house with several sensors devoted to obtaining diverse types of data, from ambient data to patient’s records. Diverse information related to patients (e.g., movement, behavioral pattern identification, activity or door closing) can be obtained from processing the collected data [3,21]. Several types of sensors can been used, e.g., motion capture, RFID, cameras, ultrasound and microphones [28]. Next, a group of projects focused on helping people to age at home, some of them funded by the Assisted Living Joint Program [29], is reviewed.

The project Aware Home [30] uses a wide variety of sensors, ranging from specifically-designed smart floors to more typical video and ultrasonic sensors, together with social robots to monitor and help older adults [31]. Another topic of interest in AAL projects over recent years has been behavior or activity monitoring. With this focus, Project House [32] presents an alternative to track the house activity using sensors like cameras or microphones, which need a signal processing computation to derive behavior conclusions. In the Gloucester Smart House project [33], a tele-care system was designed, based on lifestyle monitoring, with the pretension of continuously gathering information about the person’s activity during daily routines. The Elite care project [22] is focused on detecting diseases through the monitoring of substantial changes in the daily activity. Finally, the Ubiquitous Home Project [20], which involves the deployment of microphones at home, allows voice-based interactions between a robot and the users.

### 2.2. Wireless Acoustic Sensor Networks for Tele-Care

A WASN is a group of wireless microphone nodes spatially distributed over an indoor or outdoor environment. Its design has to take into account the scalability of the network, the delay of the acoustic signal, the synchronization of the nodes and the decision of where the computing is performed (locally or in the cloud) [4]. These systems are becoming very popular in AAL environments due the fact that they are practically non-invasive and are usually competitive in terms of cost [34]. One of the applications of sound source localization is the positioning of a person living alone [35] by means of a central system that aligns and analyzes the data coming from all the sensors. Another typical application of WASNs deployed in AAL environments is Acoustic Activity Detection (AAD) [4]. The primary purpose of AAD is to discriminate the overall acoustic events from the background noise [6], overcoming those approaches only based on energy threshold detection. Among AAD, voice activity detection plays a significant role in AAL solutions including acoustic interfaces [4].

Acoustic sensors at home can also be used, as in our proposal, for surveillance applications when taking care of the elderly or the disabled [36], and it presents huge challenges in terms of acoustic event detection. Building an acoustic-based event recognition proposal for smart homes is challenging due to the lack of higher-level comparison with environmental sounds. In [37], they worked with Mel Frequency Cepstral Coefficients (MFCC), Zero Crossing Rate (ZCR) and Discrete Wavelet Transform (DWT) features, reaching an F1 score of more than 90%. In [34], they presented an acoustic event detector system focused on a low-cost platform that records and processes the sounds at home; they presented in [34] the first results testing the accuracy, sample rate and energy consumption. In [38], they worked with acoustic signal enhancement based on independent vector analysis, in order to increase the accuracy of the system. Several experiments support the results in comparison with the baseline and, even more important, in in-the-wild situations.

Regarding the description of the applications, in [39], an acoustic fall detection system oriented toward the elderly age group living at home was described. The CIRDO project [40] was a multimodal approximation to build a healthcare system to ensure the safety of seniors and people with limited independence at home. To that effect, CIRDO implemented an audiovisual system that ran standard audio processing and video analysis tasks on a GPGPU. Despite the efforts to protect privacy and private data, the patients were still not comfortable living with a system that processed in real time the video of the home activity. Furthermore, it is quite usual to include acoustic event detection in the framework of a multimodal approach. In [7], they detailed a preliminary study focused on the recognition of predefined activities in the everyday life of elderly people using a low-power WASN, composed of audio and ultrasound sensors. Another clinically-managed AAL project was described in [18], where the goal of the proposal was to detect early stages of dementia in the elderly living at home, using the audio and video samples recorded in the house by means of egocentric cameras. In [41], up to 21 different sounds occurred in the kitchen were distinguished to define the patients’ behavior. Finally, in [42], they conducted automatic human activity recognition in a voice-controlled smart home equipped with microphones.

### 2.3. Fog Computing and Ambient Assisted Living

Given the vast amount of data that current and forthcoming AAL environments generate, existing ICT infrastructures typically struggle when processing and transmitting all the information associated with a given scenario [43,44]. Therefore, fog computing has emerged as a promising alternative to store, process and analyze data in heterogeneous environments with limited communications [45], computing, and storage facilities [46] such as the Internet of Things (IoT), AAL or eHealth [47]. In fact, the fog computing paradigm aims to decouple the data management process into two (or more) stages, which enables one to (1) conduct preliminary data processing and aggregation tasks close to where data are generated [48] (also referred to as edge computing) and (2) carry all the heavy load and intensive tasks in the cloud (also referred to as cloud computing) [44,47,49]. Hence, fog computing has resulted in a powerful tool to bridge the gap between the requirements of high performance computing for sensing environments and the features offered by clouds [50], especially in the eHealth field, where clinic-centric treatments are no longer feasible and a transition to patient-centric healthcare is required [51].

For instance, in [46], a fog data architecture was used in a tele-health application to process health data collected from wearable sensors (e.g., smart watches, ECG systems or pulse glasses). In this situation, the fog computing approach enabled practitioners to reduce the amount of data to be uploaded to the cloud [45] by more than 99%. Furthermore, in [52], a large-scale distributed infrastructure based on fog computing (i.e., smart phones and Amazon AWS instances) was proposed to conduct pervasive fall detection in stroke patients. Similarly, in [45], devices placed at the edge side of the system architecture were used to speed up the real-time data processing tasks, and a cloud platform was used to store patients’ history and metadata. Additionally, the fog computing approach can be used to reduce the typical cyber security concerns of eHealth applications by either avoiding the transfer of sensible data via third-party networks [53] or anonymizing these data before they are actually sent to the cloud [23,49]. Indeed, wearable devices deployed in an IoT fashion and exploited using the fog computing paradigm boost the potential of remote healthcare applications by enabling accurate patient monitoring, reliable data analytics and personalized services [54].

### 2.4. The HomeSound Legacy

The homeSound project [23,55] was aimed at detecting up to 14 different indoor generic sounds (e.g., dog barking, water boiling, people falling down, etc.) [56] that were related to three main categories: animals, objects or human beings. HomeSound was conceived of as a very first approach to build a general purpose platform to monitor the events that occur in an indoor environment. These events were expected to be reported to medical facilities for further analysis. This proposal raised significant interest in the AAL community because this system was able to remotely monitor patients in their home environment in a non-invasive way, by means of a low-cost platform and obtaining a reasonable accuracy close to 82%. However, the homeSound prototype cannot be directly deployed into large-scale scenarios such as the Fundació Ave Maria facilities since it still presents the following limitations:

1. Multiple acoustic sources’ cooperation [57]: Although homeSound was designed to process acoustic streams from several sources in parallel, every data stream is processed independently. Hence, the outputs of each data stream that obtain a confidence higher than a predefined threshold are serialized and sent to the medical facility. Consequently, all the events classified with low confidence are indiscriminately discarded. However, such a low confidence might be obtained due to acoustic interference (i.e., two or more events happened at the same time) or due to the fact that a given event has happened far from the microphone’s optimal range. Thus, the decision to discard the event is made without considering the output from other sources at the same time (e.g., when several microphones detect the same event with low confidence, it is likely that the event is happening) or even the historical output from the same sensor (e.g., if an event is repeatedly identified with low confidence over a short period of time, it might be worth reporting it). Therefore, the homeSound system is prone to miss low quality, yet possibly important, events.
2. Poorly-automated decision making system: The tags that the homeSound platform reports to the medical facility need to be manually analyzed by an expert team, which inevitably limits the system scalability (i.e., the more patients to be monitored, more experts analyzing 365-days/24-h data are needed) and makes the system itself prone to human error (i.e., the supervisor might miss a situation in the case of several events from different patients happening at the same time). Nonetheless, it is worth mentioning that this final layer of human supervision is usually required in this kind of AAL environment where the modest accuracy of the system may trigger false alarms [58].
3. Diversity of the events set: The homeSound platform was designed as a general purpose AAL support system without considering any specific use case. In order to demonstrate the homeSound versatility, its Learning Classifier System (LCS) was aimed at detecting a broad spectrum of events. Hence, these events have significantly different characteristics (i.e., classification features) in terms of temporal duration and frequency spectrum, which eases the duties of the event detection module (i.e., it is easier to distinguish between rain drops and a printer working than to distinguish between people talking and a patient screaming). This resulted in an optimistic overall accuracy of the classifier that hid some of its limitations (e.g., the event related to someone falling down was classified with an accuracy close to 62%).
4. General purpose training dataset: The dataset used to train the LCS of the homeSound system was composed of samples from several sources with several characteristics: noisy events, which overgeneralized the classifier model, multiple events overlapped in the same sample that are labeled as a single event, which reduced the classifier accuracy, and records sampled at different frequencies, which reduced the effectiveness of the feature set. These issues were acceptable in the context of homeSound, since it was conceived of as a proof-of-concept. However, it is reasonable to think that a better accuracy or, at least, a reduction of the sparsity in the confusion matrix may be achieved if the training dataset were refined.

The following section details the specificities of a use-case application where a large-scale AAL system needs to be implemented.

## 3. Application to FAM Residential Area

To further justify the design rationale of the proposal, this section presents the system requirements of a specific large-scale scenario where AAL services, based on acoustic event detection, need to be deployed. Additionally, a list of the most important acoustic events according to the needs of the health experts owning this use-case is presented.

### 3.1. Buildings’ Topology

FAM provides caring services to adults with intellectual disabilities to (1) proactively intervene in the areas of cognitive and physical growth and (2) improve the quality of life of patients and their families. In this regard, FAM owns different buildings, which house more than 800 patients that need to be constantly monitored. These buildings are arranged as follows:

- Residential Campus (RC): FAM manages a residential campus for patients that need widespread support. The patients live on the campus 24 hours a day, 7 days a week, 365 days a year, in several distributed houses. Hence, patients receive support from professionals in their own homes. The residential campus is more than 3000 m${}^{2}$ in three buildings and houses around 60 people who cannot live autonomously.
- Network of Houses (NH): FAM also assists a network of eight houses distributed around the village, for adults with disabilities, but who want (and are able) to live with autonomy and only need low-intensity intermittent services. These homes are for people needing a low-intensity intermittent support. The households are integrated with the community and have all necessary services. They are currently supervised by non-invasive intelligent-home equipment and security systems based on presence detection sensors, enabling limited communications with the patients.

**Figure 1:**
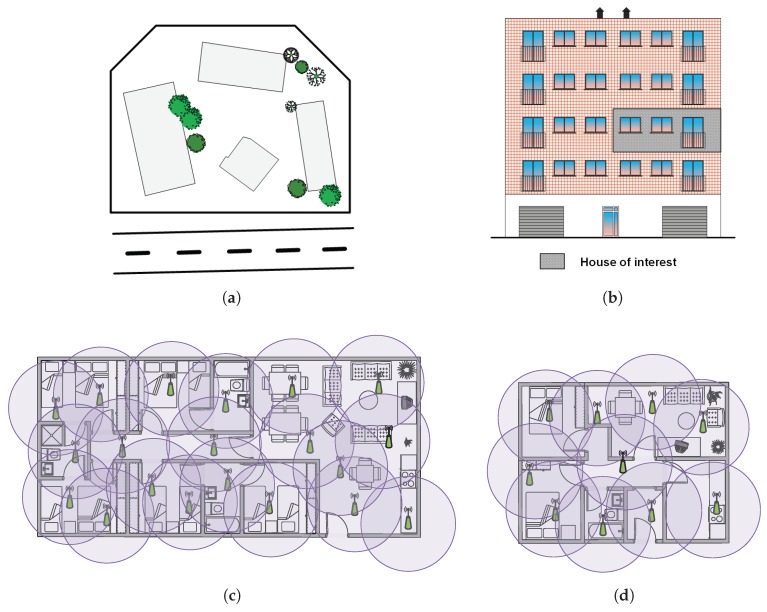
Network of housing alternatives of flats or retirement homes to be supported by the proposed AAL monitoring service and possible sensors arrangement with coverage areas. (**a**,**c**) Retirement home with several buildings/houses with common spaces (dining room, etc.) and with private facilities for the elderly or pseudo-dependent; (**b**,**d**) independent flats where elderly or pseudo-dependent people need various surveillance support.

Specifically, there are two types of houses to provide AAL services for the elderly or pseudo-dependent people. The first type, and most common so far, is a retirement home (see Figure 1a,b). In this case, the sensors need to be distributed in the common zones and, overall, in the private areas. The goal of the AAL service is to minimize the manual supervision of the patients by caring personnel, especially in private areas, so that they can devote most of their attention to actually taking care of them and not to home surveillance tasks.

The second type is a private house or flat (see Figure 1b,d). In this case, the AAL service needs to be installed in a private home, in order to remotely monitor its inhabitants and trigger alarms to the caring services. In this case, both the day and night zones will be taken into account for the deployment of the sensors, since the predefined alarms require surveillance in both areas.

As observed, the size of the scenarios is diverse, which is nowadays one of the challenges of the implementation of the pilot. There are two different scenarios; the residence is a big building (two floors, at least 12 different rooms, taking into account the bathroom, kitchen, living room and dormitories), and the flats have variable sizes (from 3–6 rooms). For this reason, the first part of the proof of concept assumes that the system is only in the living room of the houses and the residences, in order to make the tests more homogeneous. The second stage (in the future) will study the optimum number of sensors to be deployed in each scenario depending on its size and room distribution.

Hence, the requirements in terms of system architecture of the proposed platform are as follows:

1. Large-scale monitoring: Patient home locations can be distributed all over a medium-size city and have no possibility of sharing information apart from the Internet (i.e., there are no dedicated communication networks).
2. Scalability: An arbitrary number of houses (and patients) under supervision and monitoring can be added or removed at will.
3. Reliability and fault tolerance: All of the patient facilities need to be monitored constantly in order to trigger an alarm as soon as an emergency situation is detected.
4. Monitoring heterogeneous scenarios. Sensors will be deployed in home environments at both the residential campus and the network of houses. The system should be flexible enough to tolerate different environments, sizes and number of sensors (typically proportional to the dimensions of the house), while avoiding black coverage zones in any of the facilities.

Therefore, given the spatial heterogeneity of the FAM facilities, acoustic event detection [23] offers a good trade-off between deployment costs, communication bandwidth consumption and accuracy. The following section details the considered acoustic events to be detected according to FAM health experts.

### 3.2. Acoustic Nature of Events

There are many acoustic events that are likely to trigger an alarm in an apartment or room where an elderly or pseudo-dependent person lives. For the sake of this proposal, the FAM caregivers and experts have proposed four lines of action that contemplate up to nine different types of sound (i.e., door knocking, scream, people talking, silence, door closing, telephone, television, door bell and glass breaking), whose temporal analysis can determine whether their associated event can be classified to give a warning. These sounds can be grouped into the following categories:

1. Door bell or phone ring: An unanswered doorbell or phone ringing over a long period of time is considered important enough to activate the alarm. This means that there is nobody to answer at home or that the person who is in the home is not in a condition to answer.
2. Presence of people at home besides the patient(s): The presence of many people at home or in a certain room is a potential risk. Unauthorized persons may have entered the room, and the patient may find him/herself in an intimidating situation. It might not be a risky situation, but the alarm must be raised as a preventative measure.
3. Patient shouting: The patients’ screams are always a sign of alarm. They can be caused due to not being well, by suffering some anxiety or panic attack or by any other possible emergency situation (fire, theft, etc).
4. Activity at home after hours: Voices, television, music or any other sign of activity after hours is also cause for alarm. Being awake and active during the night can indicate disorientation or any other type of emergency at home.

**Figure 2:**
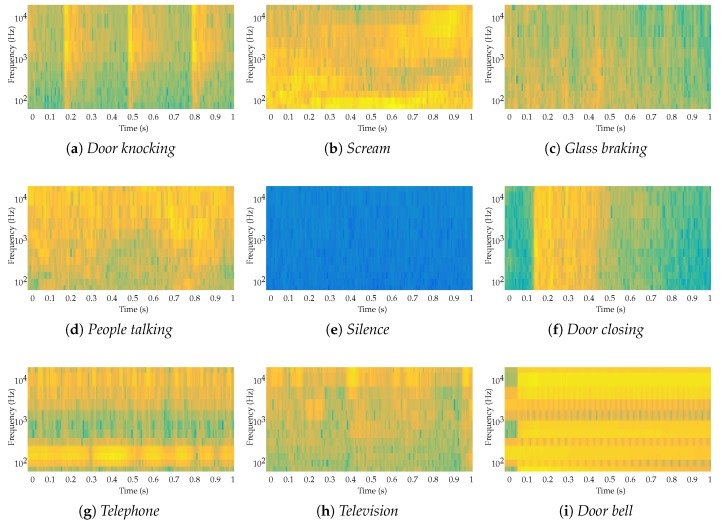
Spectrograms of the nine types of sound.

The spectral representation of all nine acoustic events are depicted in Figure 2. Although the frequency occupation range is pretty similar, there are subtle differences that enable us to distinguish them. For instance, it can be observed that the events *people talking*, *television* and *scream* have similar distributions: they all come mainly from the human voice; but, the *television* has stronger components at low frequencies; *scream* has stronger components at high frequencies; and *people talking* has stronger components at medium frequencies. In a similar way, the *door bell* event has strong components at both medium and low frequencies. On the contrary, the *silence* event (i.e., ambient noise) lacks strong components at high frequencies. Furthermore, there is a great difference between the repetition period of *door knocking* (i.e., ∼0.3 s) and the *telephone* (i.e. ∼0.02 s) events. Additionally, this repetition period can be used to differentiate the *door knocking* event from the *door closing*, which show similar frequency patterns (i.e., abrupt frequency increase and smooth frequency decrease), but the *door closing* lacks repetitions. The *glass breaking* event also evolves its frequency distribution as time passes.

Therefore, the appropriate automatic system to identify these events with a reasonable accuracy has to consider both their frequency distribution and their temporal patterns, while the latter in some cases provide crucial information to distinguish between similar pairs.

## 4. System Architecture

Extending the achievements obtained in indoor environments [23] to larger domains such as residential areas requires (1) improving the communications network to support data streams’ processing from multiple sources in real time; (2) refining the feature extraction and signal processing techniques to avoid network bottlenecks [59] (finding a good trade-off between local data processing and the amount of data to be deferred [60]) and (3) enhancing the acoustic event detection system to provide reliable outputs [61] with minimal human interaction. This section describes the proposed system architecture to address the AAL requirements at FAM facilities considering the aforementioned three design requirements.

Wi-Fi networks offer a convenient trade-off between deployment and infrastructural costs, power consumption and available bandwidth [62], especially in the context of AAL solutions. Therefore, they are often used to connect the sensing layer (i.e., data acquisition in an indoor environment) with the analytic/storage/processing/computing layer of AAL solutions [63]. Usually, [46,49,50,51,52,53,54], the computing layer is not physically located close to the patient facilities (e.g., cloud computing infrastructure); hence, the Internet is used to link both domains, which reduces the overall available bandwidth, makes the system prone to cyber security threats [64] and adds a considerable delay to the event detection process. It is worth considering that the negative effects of these issues (i.e., bandwidth, cyber security and delay) grow with the number of patients and/or area to be monitored since their associated sensed data also grow accordingly, as is the case of residential areas such as the FAM facilities under study.

**Figure 3:**
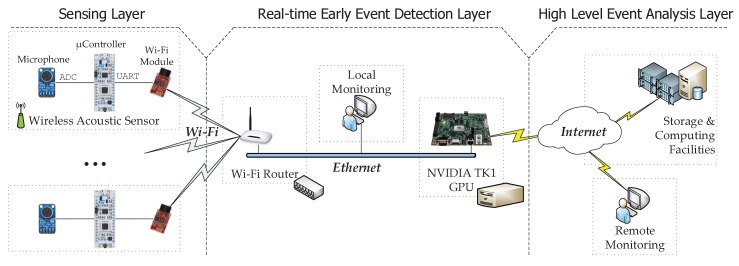
Network topology of the proposed system.

Therefore, we propose the fog-inspired [50] distributed architecture depicted in Figure 3 to (1) support data collection from multiple sources; (2) provide a real-time early event detection layer and (3) refine the event detection process with a high-level event analysis layer. Hence, the proposed architecture is divided into three layers:

1. Sensing layer: It is composed of all the wireless acoustic sensors that are deployed over the area where the patients need to be monitored. Hence, every sensor is committed to (1) sampling the raw audio at 44.1 ksps; (2) extracting the audio features from the acoustic samples (i.e., build a features vector) to avoid flooding the network with acoustic data streams and (3) sending these features to the wireless hub. All this is achieved by means of inexpensive hardware (around 20 €): an electric microphone with the amplifier MAX9814 breakout board, a Nucleo 32 development platform with the STM32L432KC ARM cortex-M μController and a Wi-Fi module based on the ESP8266.
2. Real-time early event detection layer: Every acoustic features vector received from the sensing layer is analyzed in an embedded GPGPU NVIDIA Jetson TK1 [27]. This GPGPU is a convenient design choice that enables the system itself to analyze several data streams in parallel [23]. In this regard, this GPGPU contains a trained Artificial Neural Network (ANN) model. The trained ANN running in exploitation mode provides a weighted label vector that will be sent to the high-level event analysis layer. Each component of the vector corresponds to the probability of each event, also known as classification confidence. This can be best seen as a preliminary notion of the event that might have happened (i.e., taking the component with the highest value of the vector), since it does not consider the time domain of the events (e.g., multiple consecutive vectors indicating a door closing might mean that the actual event is door knocking). Therefore, users should understand that the provided information by this layer is not reliable at all, and thus, further actions taken upon the labels of a single stream should be prevented. Alternatively, by making early decisions at this layer, which might be useful for events that require immediate assistance such as screaming or glass breaking, users can take advantage of the fact that WASs cover overlapped areas (see Figure 1), and thus, multiple streams can be analyzed concurrently in real time to reliably find whether an event has been detected at different adjacent locations.
3. High-level event analysis layer: The purpose of this second event classification level is two-fold. On the one hand, the system analyzes the acoustic events according to their context (i.e., the events that happened within a few acoustic frames of each other). Therefore, rare events occurring in a single frame might be filtered (e.g., people talking). On the other hand, it also takes into account the data streams from adjacent locations. In this way, those events classified with low confidence, but identified at different WASs, can gain relevance at this second classification layer. To achieve this goal, the frames collected at the real-time early event detection layer are concatenated and compared against a large case memory [65]. Finally, this layer generates user-defined alarms on the detected events. These alarms are defined by means of heuristic methods (e.g., trigger an alarm if *television* is detected between 3 a.m. and 6 a.m.).

To sum up, the data computation process has been split as in typical fog computing systems to enable real-time feature extraction and alarm triggering: WASs extract the audio features; An initial acoustic event classification and detection is conducted by the local GPGPU; and finally, the classification process is refined using the CBR deployed at the cloud infrastructure, which drives the alarm triggering process.

**Figure 4:**
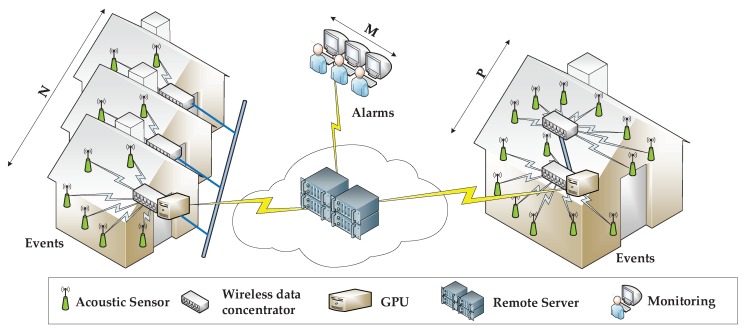
Proposed system architecture particularized for the Fundació Ave Maria use-case.

As shown in Figure 4, the proposed system architecture fits the specificities of FAM facilities. A wireless hub is deployed in every building to provide indoor Wi-Fi connectivity. Furthermore, several wireless hubs can be deployed in the same building or in adjacent buildings, as is the case of the FAM residential campus, and interconnected by means of Ethernet to extend the Wi-Fi coverage area. Each WAS uses its associated Wi-Fi network to send data to the GPGPU. Subsequently, each GPU sends the output of the early event detection layer to the remote servers deployed on the cloud by means of the Internet. Caregivers can monitor the alarms and the status of the patients by either connecting to the remote servers or to local GPGPUs.

The following section discusses the audio features used by the acoustic event classification system and how their extraction and processing are related to the proposed system architecture.

## 5. Acoustic Event Classification

**Figure 5:**
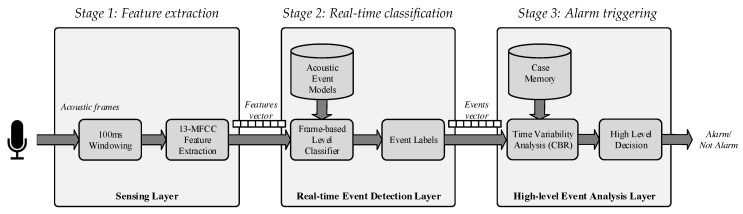
Block diagram of the proposed acoustic event classification system.

The proposed audio event detection algorithm consists of three stages that directly map to the three layers proposed in Figure 3. As shown in Figure 5 these stages are the following: feature extraction, real-time classification and alarm triggering. Each stage is described as follows.

Stage 1. Feature extraction: This is conducted at the sensing layer in Figure 3 and consists of a signal processing procedure to (1) find a set of coefficients (i.e., features) that characterize the audio samples and (2) reduce their dimensionality. Specifically, we have used the same approach as in [23] that consists of using the first 13 MFCC to characterize the acoustic samples. There are two main reasons for selecting MFCC: (1) they are a de facto standard for use in the Acoustic Event Detection (AED) community [4,37] and (2) they can be computed efficiently in real time [23], which is an important issue to take into account for the problem to solve, with 24 h, seven days a week of data collected. Therefore, the 13-component vectors of MFCCs are computed at this stage using a 100-ms window with an overlapping factor of 50% applied to the audio input.
Stage 2. Real-time classification: This is conducted at the real-time event detection layer in Figure 3 and consists of an ANN deployed over a GPGPU that takes the 13 MFCC coefficients computed at the sensing layer, compares them against the ANN model and outputs a nine-component vector (i.e., one component for each possible acoustic event). The ANN is computationally inexpensive using a GPGPU since several arithmetic products can be done in parallel (see Section 6.2). Additionally, the parallel processing capabilities of the GPGPU enable practitioners to run several ANNs concurrently to reduce the acoustic event classification delay.
Stage 3. Alarm triggering: This is conducted at the real-time event detection layer in Figure 3 and consists of a CBR system [65] and a high-level decision module to decide whether or not to trigger an alarm. More specifically, the nine-component vectors are concatenated in a circular buffer of 900 buckets, which corresponds to the detected events at the previous stage of the last 10 sec. To take advantage of the sensor redundancy, the circular buffers associated with the data streams from WASs deployed at adjacent locations are averaged Then, the whole circular buffer is compared against a large case memory (synthetically populated) to output a nine-component binary vector, where each component indicates whether the event has happened or not. Once the binary vector is generated, it is analyzed (i.e., high level decision module) by means of a set of user-defined heuristics and rules that, according to the vector values and time of day, define those situations in which an alarm must be triggered. This heuristics module also has a memory component to contemplate those situations in which medium-term repetitions are meaningful.

This process runs in parallel for each data stream associated with the WASs deployed at the FAM facilities. Hence, for every new frame (i.e., every 100 ms), Stages 1 and 2 recompute their output. Although Stage 3 decides whether or not to trigger an alarm every 10 s, it also recomputes its output every 100 ms. In this way, caregivers can examine the patient status in real time.

## 6. Preliminary Evaluation and Discussion

After the design, development and proposal of the platform to support AAL (i.e., conduct acoustic event detection) in residential areas, this section aims to share several preliminary classification results, as well as the main lessons learned on the conception of this proposal and the discussion of future work and challenges.

### 6.1. Preliminary Audio Classification Tests

To assess the feasibility of the proposed approach, we have analyzed the behavior of the proposed system at every stage in Figure 3 since data are collected until alarms are generated.

First, the ANN of the real-time early event detection layer has been trained using back-propagation [66] with a dataset of 7116 s, composed of the nine sounds detailed in Section 3 obtained from public repositories and annotated as done in [67]. As some of these events are unusual (e.g., *glass breaking*, *scream*, *door knocking*), physically recording them at FAM facilities to get representative samples is barely feasible, which results in a highly imbalanced dataset.

To obtain statistically-significant insights, the ANN has been run 10,000 times, obtaining results for a 10-fold cross-validation [23,68]. For each data fold, 70% of the audio files from each class are used to train the ANN, and the remaining 30% are used to test it, obtaining an overall accuracy of 85.4% and an F1 score of 71%, which despite being an early and unreliable event detection layer, has a performance similar to the final accuracy of existing approaches [23]. To further understand the behavior of the ANN, the confusion matrix shown in Table 1 has been built. It can be seen that the most common classification error is between several pairs of classes *door knocking* and *door closing*. This situation is very common, as both events are practically the same considering a 100-ms window, and the only difference is their repetition pattern (see sub-figures *a* and *f* in Figure 2). Furthermore, it is worth mentioning that the probability of missing an acoustic event (i.e., classifying an actual event as *silence*) is considerably low (i.e., lower than 0.5%) as shown in the *silence* column in Table 1. This ensures an initial high reliability in the early detection of acoustic events despite having a moderate accuracy in distinguishing the event.

Therefore, the capability of the high level event analysis layer to correct the classification errors of the ANN is illustrated in what follows, as typically done in similar works [56,71,72]. In this regard, a synthetic audio data stream containing all the possible events has been built to model a typical day of an inhabitant at the FAM facilities in 200 s. Acoustic samples used for the sake of this experiment have been carefully selected to stress the strengths and weaknesses of the classifier. The MFCCs of this acoustic data stream are computed at the sensing layer and sent to the ANN classifier, which delivers its output to the high-level event analysis layer.

**Figure 6:**
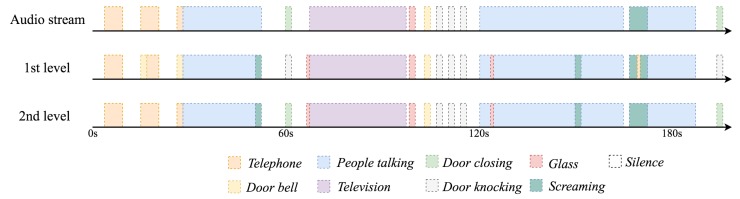
Temporal evolution of the first level (early event real-time detection) and second level (high level event analysis) classifiers. The 10-s delay of the high-level event analysis layer has been removed to ease the output comparison between two layers.

The output of both acoustic event detection stages when processing this data stream is shown in Figure 6. First, someone calls the patient (i.e., *telephone* event), and the telephone rings three times before it is answered. Although the first level of the classifier may confuse some parts of the telephone ringing with a *door bell* (by just looking at the MFCCs from a 100-ms window), the second level of the classifier is able to identify it as a *telephone* call considering the temporal pattern of the signal. Next, the patient picks up the phone and talks (i.e., ion confuses part of the phone conversation with a *scream*, and the second level of the classification also fails to correct it. However, as the patient keeps talking on the phone, this *scream* would not generate an alarm. A few seconds after the phone call, a door is closed (i.e., *door closing* event). The first level of the classifier confuses this event with a *door knocking*, but the second level of the classifier is able to correct it by reasoning that there are no more fragments classified with the same label in a short period of time. Later on, at Second 120, a conversation (i.e., *people talking* event) starts and produces two ghost events (i.e., events that have nothing to do with the actual sound) that the second level of the classifier is not able to correct. No emergency alarms are generated due to the fact that an abrupt change in the context of the scenario has not been perceived (i.e., the system assumes that everything is under control).

Finally, after the 10,000 runs previously carried out, but now considering this second classification stage, which adds a decision delay of 10 s, the classification accuracy of the proposed system increases up to 93.27%, and the F1 score increases up to 88.14%. Note that although Figure 6 only shows the event with the highest confidence in the first layer, the whole vector is still delivered to the high-level event analysis layer. Hence, if an additional WAS is deployed and examines a noised version (i.e., adding 1 dB of additive white Gaussian noise) of the same data stream (to emulate two adjacent WASs) the overall classification accuracy increases up to 94.6%, and the F1 score increases up to 90.58%.

### 6.2. Scalability of the Proposed Infrastructure to Support Ambient Assisted Living Services

Scalability is one of the key challenges in the design of an AAL platform to support tele-care services in large-scale scenarios. Existing approaches that integrate multiple heavy data streams (e.g., video) require expensive, and often ad hoc, communication infrastructures [73], which might limit the performance of the system when multiple data streams need to be analyzed concurrently. Therefore, the proposed system architecture inspired by fog computing systems has been conceived of with two main design goals: (1) reduce the amount of data to be transferred (i.e., moving computation is less expensive than moving data [48]) and (2) take advantage of existing infrastructures in typical use-case scenarios (e.g., Wi-Fi and Internet connectivity) to reduce deployment expenses. Hence, the platform scalability is further discussed and sketched in what follows.

First and foremost, the amount of raw data to be transferred at the sensing layer between every WAS and the Wi-Fi router can be computed as:

(1) $$Bytes\;to\;be\;transferred_{WAS}=\frac{13\;MFCCs}{WAS*Window}*\frac{1\;Window}{100\;\mathrm{ms}*0.5}*\frac{8\;bytes}{1\;MFCC}=2\;080\;\frac{bytes}{second*WAS}$$

However, these data need to be framed; hence, considering minimal UDP headers over Ethernet (i.e., 52 bytes), the real throughput of a WAS at the sensing layer is 3120 Bps (24.375 Kbps). Although this is a small number compared to regular Wi-Fi speeds of current home devices (e.g., 54 Mbps), theoretically enabling one to connect a few thousand WASs to the same Wi-Fi router, it must be considered that: (1) most of the standard Wi-Fi routers cannot accept more than ≈255 devices; (2) the data stream nature of this application (i.e., a new frame is generated every 50 ms by each WAS) reduces the available Wi-Fi throughput [74] considerably (e.g., 54 Mbps available bandwidth shall be limited to ≈21.6 Mbps); and (3) the delay increases with the number of the WAS connected to the Wi-Fi network. Nevertheless, the preliminary analyses conducted have shown us that we can safely connect up to 60 WASs without perceiving a performance degradation, which generates a constant data flow of ≈1.43 Mbps per Wi-Fi access point.

Hence, more access points can be added to the network until exhausting the available bandwidth of the Ethernet wired connection (e.g., 100 Mbps or 1 Gbps) without neglecting the traffic generated by the GPGPU, which can be computed as follows:

(2) $$Bytes\;to\;be\;transferred_{GPU}=\frac{9\;labels}{WAS*Window}*\frac{1\;Window}{100\;\mathrm{ms}*0.5}*\frac{4\;bytes}{1\;label}=720\;\frac{bytes}{second*WAS}$$

Considering again minimal UDP headers over Ethernet, the real throughput associated with a WAS generated by the GPGPU is 1760 Bps (13.75 Kbps). Furthermore, it must be considered that the number of WASs will be limited by the available bandwidth of the Internet connection. For instance, taking an average residential 2-Mbps ADSL connection and a 100-Mbps LAN, we could theoretically support up to ≈2449 WASs, which at the same time would require up to 40 Wi-Fi access points.

As far as the GPGPU is concerned, the computational cost of the proposed ANN can be approximated to 14.2 KFLOPS per WAS, which is also smaller than the computational capabilities of the NVIDIA GPGPU that is in the order of 13.6 GFLOPS per core according to [75].

Overall, it can be seen that the Internet connection is the scalability limiting factor of the proposed architecture. However, this can be easily addressed by adding more Internet connections to the facility of interest when needed.

Additionally, as the sensing layer of the proposed system is deployed over casual infrastructures (i.e., generic purpose home Local Area Networks (LAN)), it is worth analyzing its fault tolerance:

1. Faults at the sensing layer (e.g., a WAS stops running or loses connection with the Wi-Fi router) are not critical at all thanks to the redundancy of the WASs. However, if the Wi-Fi router stopped working, all data associated with its associated WASs would be lost. In this situation, the GPGPU would notice the absence of data streams and would trigger an alarm. Alternatively, the WASs could use the data connection of a standard cell phone to reach the GPGPU through the Internet, given the low amount of data (i.e., 24.375 Kbps) to be transferred (see Equation (1)).
2. Faults at the GPGPU can be addressed by redirecting the data streams to another GPGPU through the local Ethernet (as long as there are more GPGPUs in the same LAN) or through the Internet.
3. Faults in the Internet connection may isolate a given building. However, the data connection of a standard cell phone could also afford the generated traffic, given the low amount of data (i.e., 13.75 Kbps/WAS) to be transferred (see Equation (2)). Additionally, real-time data would be always available for all the caregivers connected at the same LAN as patients.
4. Faults at the high-level event analysis layer are unlikely given that the cloud services provider ensures a predefined degree of QoS.

Furthermore, this distributed approach contributes to ensuring the patients’ cyber privacy: raw acoustic data streams are never broadcast through the network. Instead, the acoustic features and detected events are encrypted whenever they have to cross any communications network. This, together with the non-invasive nature of the acoustic event detection, makes the proposed platform very suitable for patients reluctant to wear unaesthetic and often uncomfortable devices [73,76].

To sum up, taking Figure 4 as a reference, the system may be able to scale as follows: (1) the number of adjacent buildings *N* can easily grow as long as there is enough bandwidth at the Ethernet link (recall that few features are generated per acoustic data stream); (2) the number of disseminated homes *P* (i.e., the number of GPGPUs that deliver data to the remote servers) can also grow according to the capabilities of the cloud infrastructure on elastically adapting to the *P* buildings’ demands. Similarly, the number of users *M* monitoring the patients could also grow as long as the cloud and the Internet can tolerate more connections (i.e., recall that only label events and time-stamps are exchanged at this layer).

### 6.3. Signal Processing Challenges

In this first approach to the design of an acoustic AAL system for residential environments, only up to two sensors have been taken into account for the analysis of the results to measure the classifier accuracy. The signal processing and machine learning algorithms tested to conduct this first approach have been designed to support the proof of concept, assuming that it is a crucial part to be improved in the future. In this sense, there are two major challenges for the processing part of the acoustic signal.

The first one corresponds to the selection of both the type and the number of coefficients of the feature extraction algorithm. In this work, this phase has been carried out using a baseline, the MFCC [77] and 13 coefficients, in a coherent way with past works [23]. To ensure that the feature extraction performs the best it can, further tests should be conducted in two directions: test the distribution of hits using other feature extraction algorithms, such as Gammatone Cepstral Coefficients (GTCC) [78], or narrowband coefficients [79], or any other proposal that can be found in the literature [80]. This exhaustive test should be expanded with a detailed study of the affect of the number of final coefficients used in direct relation with the results in terms of accuracy. This double study would allow us to see if there are many variations in the quality of the hits according to the type of sound. One could even consider working with several types of coefficients, proposing a hybrid acoustic signal processing block.

The second challenge is to address the processing of multiple acoustic data streams as a whole. In this regard, it is essential that the distribution of the sensors in the homes be done in a fully-redundant manner, that is all points must be covered by at least two sensors. In this proof of concept, we have seen that this redundancy increases the classifier accuracy (see Section 6.1). However, we believe that a more intelligent system, rather than the average one proposed in this work, would increase the system performance. In this sense, the high-level event analysis layer should be refined in order to make it able to decide whether or not to trigger the alarm or not conditioned on what the sensor(s) deployed next to it have sensed, which would be similar to the reduced variable neighbor search proposed in [43]. This would require (1) prior knowledge of the exact location where the sensors are deployed and (2) a reconstruction of the case memory accordingly.

Finally, the accuracy requirements of the entire system should be taken into account assuming the limitations of the acoustic event detection framework. In some specific places (e.g., kitchen or dining room) where the patient privacy concerns can be relaxed, other sensors could be installed, as suggested in [81], which would make the proposed system process parts of video streams if there were the possibility of an emergency. However, the accuracy of the proposed system versus the privacy of the inhabitants (already discussed in this work)is always an element to take into account for a final design.

### 6.4. Multi-Layered Acoustic Event Detection Process

To improve the accuracy of existing approaches to provide AAL services based on acoustic event detection [23], we have (1) used a different learning classifier system and (2) split the classification process into two stages: the first level classifies 100-ms frames, and the second level classifies 10-s frames. The classification algorithm used at the second level (i.e., CBR) enables us to build a human-readable model (i.e., the case memory can be easily read and populated by expert caregivers), but at the price of consuming a large amount of memory. This approach enables us to easily debug the triggered false alarms and include new situations quickly. However, if a larger dataset were available, the obtained results could be improved by using a long short-term memory network [82], which would enable us to build a more robust, yet non-human readable, model for the second classification layer.

Another issue with the obtained dataset is class imbalance. It is well known that classifiers do not perform properly when there are significant differences in the number of instances per class. It is actually very difficult to obtain a considerable number of meaningful acoustic samples of the events under interest; for the sake of this work, more than 20 hours of audio were analyzed to build the 7116-s dataset. Therefore, we think that it would be useful to (1) record the events where the system is going to be deployed (e.g., the sound of a wood door is not the same as the sound of a glass door); (2) conduct data augmentation techniques to enrich the variability of the samples from each class [83] and (3) add synthetic samples to cover those situations where more than one event happens at the same time.

## 7. Conclusions

This article presents the proof of concept of a distributed ambient assisted living platform that aims to trigger alarms based on acoustic detection of specific events, in the framework of several living environments, such as residential areas, residences or private homes. More specifically, the design and implementation of a scalable architecture inspired by the fog computing paradigm that splits the sensing, processing and alarm triggering layers has been proposed, obeying an initial event recognition proposal by FAM in their interest areas. This platform has been conceived of to address the ever growing needs in terms of area coverage and response time of modern AAL systems. To further stress this situation, the requirements of the Fundació Ave Maria non-profit organization have been presented, and the proposed platform has been adapted to address them.

On top of this distributed architecture, an automatic acoustic event classification system has been deployed. The classification process has been split into two stages. The first stage is based on an ANN and performs a real-time acoustic event detection with an overall accuracy of 85.4% and an F1 score of 71%. The second classification stage considers the temporal evolution of the detected events in a 10-second interval by means of a CBR algorithm and a set of heuristics, which enables the system to increase its overall accuracy up to 94.6% and its F1 score up to 90.58%, when two concurrent acoustic data streams are considered.

As a result, the proposed general purpose proof-of-concept presents a reasonable accuracy for the detection of the events of interest in large AAL scenarios, despite the limited training dataset, with an assumable computational overhead and limited architectural costs. In fact, the system accuracy could be improved if more sophisticated data mining techniques such as data augmentation or transfer learning were applied conveniently. Actually, the model of the ANN and, thus, the CBR should be fine-tuned once the system is deployed according to the room, building and environmental characteristics. Hence, this prototype can be considered as a baseline to build AAL services.

As future work, we plan to record a new dataset in a real environment with multiple sensors, to maximize the performance of the proposed prototype. We also plan to improve the detection of acoustic events considering all the sensors of the network as a whole.

## Figures and Tables

**Table 1:** Accuracy at the real-time early event detection layer with the following ANN configuration: network topology: full mesh; number of layers: 5; number of neurons per layer: 100, 70, 50, 30, 10; hidden layers’ activation function: rectified linear unit [69]; output layer activation function: softmax [70]. On the top, confusion matrix. On the bottom, detailed accuracy by class computed as one-vs.-all.

Predicted Class										
		***Door knocking***	***Screaming***	***People talking***	***Silence***	***Door closing***	***Telephone***	***Television***	***Door bell***	***Glass***
Actual Class	*Door knocking*	93.21%	0.01%	0.58%	0.10%	2.03%	0.01%	3.39%	0.19%	0.48%
	*Screaming*	4.63%	79.01%	2.23%	0.12%	1.32%	6.57%	2.41%	2.02%	1.69%
	*People talking*	0.72%	5.43%	91.67%	0.23%	1.04%	0.23%	0.03%	0.28%	0.37%
	*Silence*	1.87%	0.46%	3.85%	69.23%	0.19%	0.21%	0.40 %	0.71%	23.08%
	*Door closing*	**85.71%**	2.98%	4.23%	0.49%	**4.17%**	0.08%	0.68%	0.19%	1.47%
	*Telephone*	4.13%	2.33%	0.04%	0.02%	1.94%	80.83%	0.39%	4.49%	5.83%
	*Television*	0.23%	0.12%	4.88%	0.06%	0.19%	0.16%	94.12%	0.20%	0.04%
	*Door bell*	0.77%	0.68%	0.11%	0.34%	0.21%	**8.42%**	1.05%	**67.37%**	**21.05%**
	*Glass*	1.82%	0.88%	0.02%	0.02%	0.28%	0.65%	0.06%	0.38%	95.86%
		**Sensitivity**	**FPR**	**Precision**	**Specificity**	**F-Measure**	**MCC**	**AUC**	**PRC Area**	
Actual Class	*Door knocking*	0.9321	0.0305	0.6973	0.9695	0.7978	0.7899	0.9508	0.6174	
	*Screaming*	0.7901	0.0132	0.6051	0.9868	0.6853	0.6826	0.8885	0.5925	
	*People talking*	0.9167	0.0203	0.8221	0.9797	0.8668	0.8539	0.9482	0.5473	
	*Silence*	0.6923	0.0014	0.9965	0.9986	0.817	0.7628	0.8454	0.3479	
	*Door closing*	0.0417	0.0075	0.0606	0.9925	0.0494	0.0411	0.5171	0.4906	
	*Telephone*	0.8083	0.0175	0.9093	0.9825	0.8558	0.8288	0.8954	0.4495	
	*Television*	0.9412	0.0071	0.8756	0.9929	0.9072	0.9028	0.9671	0.5328	
	*Door bell*	0.6737	0.0138	0.8828	0.9862	0.7642	0.7421	0.8300	0.3954	
	*Glass*	0.9589	0.1346	0.3370	0.8654	0.4987	0.5244	0.9122	0.8109	

## Abbreviations

The following abbreviations are used in this manuscript:

AAD	Acoustic Activity Detection
AAL	Ambient Assisted Living
AED	Acoustic Event Detection
ANN	Artificial Neural Network
AUC	Area Under the Curve
CBR	Case-Based Reasoning
DH	Disseminated Home
FAM	Fundació Ave Maria
FPR	False Positive Rate
GPGPU	General Purpose Graphics Processing Unit
GRITS	Grup de recerca en Internet Technologies and Storage
GTCC	Gammatone Cepstral Coefficients
GTM	Grup de recerca en Tecnologies Mèdia
ICT	Information and Communication Technologies
IoT	Internet of Things
LAN	Local Area Network
LCS	Learning Classifier System
MCC	Matthews Correlation Coefficient
MFCC	Mel Frequency Cepstral Coefficients
PRC	Precision-Recall Curve
QoS	Quality of Service
RC	Residential Campus
t-SNE	*t*-distributed Stochastic Neighbor Embedding
UDP	User Datagram Protocol
WAS	Wireless Acoustic Sensor
WASN	Wireless Acoustic Sensor Network
WSN	Wireless Sensor Network
UART	Universal Asynchronous Receiver-Transmitter
